# Sensitivity Analysis of Component Parameters in Dual-Channel Time-Domain Correlated UWB Fuze Receivers Under Parametric Deviations

**DOI:** 10.3390/s25165065

**Published:** 2025-08-14

**Authors:** Yanbin Liang, Kaiwei Wu, Bing Yang, Shijun Hao, Zhonghua Huang

**Affiliations:** School of Mechatronical Engineering, Beijing Institute of Technology, Beijing 100081, China; liangyanbin@bit.edu.cn (Y.L.); 3120205163@bit.edu.cn (K.W.); 3120215164@bit.edu.cn (B.Y.); 3120215165@bit.edu.cn (S.H.)

**Keywords:** ultra-wideband (UWB), fuze receiver, tolerance, sensitivity analysis

## Abstract

In ultra-wideband (UWB) radio fuze architectures, the receiver serves as the core component for receiving target-reflected signals, with its performance directly determining system detection accuracy. Manufacturing tolerances and operational environments induce inherent stochastic perturbations in circuit components, causing deviations of actual parameters from nominal values. This consequently degrades the signal-to-noise ratio (SNR) of receiver outputs and compromises ranging precision. To overcome these limitations and identify critical sensitive components in the receiver, this study proposes the following: (1) A dual-channel time-domain correlated UWB fuze detection model; and (2) the integration of an asymmetric tolerance mathematical model for dual-channel correlated receivers with a Morris-LHS-Sobol collaborative strategy to quantify independent effects and coupling interactions across multidimensional parameter spaces. Simulation results demonstrate that integrating capacitors and resistors constitute the dominant sensitivity sources, exhibiting significantly positive synergistic effects. Physical simulation correlation and hardware circuit verification confirms that the proposed model and sensitivity analysis method outperform conventional approaches in tolerance resolution and allocation optimization, thereby advancing the theoretical characterization of nonlinear coupling effects between parameters.

## 1. Introduction

Fixed-distance fuze is an electronic device that emits trigger signals at preset distances via radio frequency (RF), laser, UWB, or millimeter-wave (MMW) technologies [1]. The UWB proximity fuze detects and identifies target distance, velocity, and detailed signatures by transmitting and receiving carrier-free ultranarrow pulses. As a core technology in modern precision-guided munitions, its widespread adoption originates from three key advantages: sub-nanosecond pulse width, centimeter-level range resolution, and exceptional anti-jamming capabilities [2,3]. Through time-domain correlation processing of target echoes, it effectively suppresses environmental clutter while significantly enhancing height-of-burst control precision and battlefield adaptability in complex electromagnetic environments. This fulfills precision terminal ranging requirements for diverse missile and bomb systems [4,5].

The UWB fuze system incorporates four essential functional modules: pulse transmitter module, antenna module, correlation receiver module, and signal processing module [6].

As front-end signal acquisition units, the performance of UWB fuze receivers critically determines munition system efficacy and operational reliability [7]. Receivers’ precision in extracting weak target echoes from high-intensity background noise governs ranging accuracy and target detection success rates. Conventional fuze receivers typically employ a single-channel correlation receiver architecture. While effective external noise suppression techniques have been established [8,9], research on intrinsic mechanisms for mitigating internal thermal noise within the receiver systems remains understudied. These receivers exhibit inherent susceptibility to thermal noise interference [10], constituting a critical performance bottleneck. This phenomenon originates from the random thermal motion of electrons within conductive components (e.g., capacitors, resistors) during temperature fluctuations [11], generating stochastic current pulses manifested as spontaneous voltage fluctuations across resistive elements [12]. Critically, single-channel architectures lack inherent noise cancelation capabilities despite employing narrow-pulse sampling strategies, constraining the output signal-to-noise ratio (SNR). This limitation directly compromises weak target detection fidelity and elevates risks of false alarms and missed detections in complex electromagnetic environments [13].

**Figure 1:**
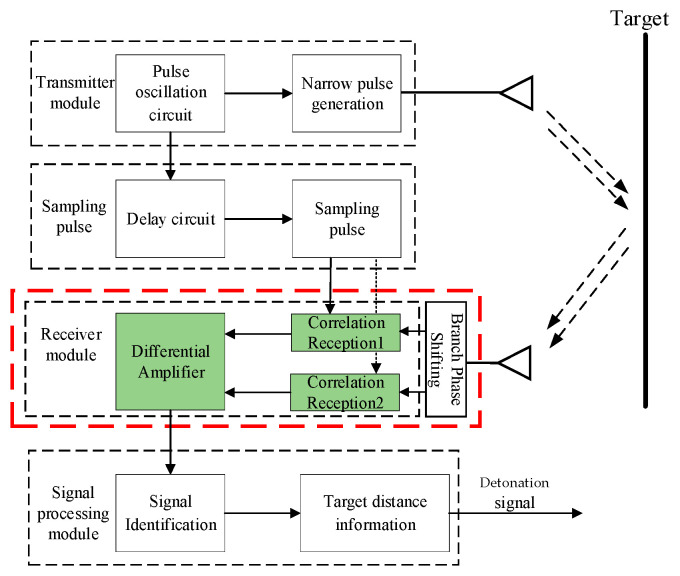
Dual-channel time-domain correlated UWB fuze block diagram.

Building upon the original receiver circuit structure [4], this work thus proposes a dual-branch differential receiver architecture, as shown in Figure 1, to overcome the SNR limitations inherent in single-branch correlation reception for UWB fuzes. As shown in the internal structure within the red frame in Figure 1, the design incorporates a counter-phase branch with strict 180° phase shifting into the conventional single-branch framework, leveraging a differential amplifier to achieve common-mode noise rejection and differential-mode signal amplification, thereby enhancing signal integrity transmission to subsequent modules. The intrinsic advantage of the dual-path correlation differential circuitry lies in the inherent phase-inversion and amplitude–symmetry of its input signals—processing two RF signals maintaining strict 180° phase opposition and equal amplitude through a differential structure enables circuit-internal thermal noise cancelation while constructively superimposing signal energy, thereby enhancing output SNR.

The noise suppression efficacy of this architecture, as shown in Figure 1, fundamentally depends on ideal symmetry assumptions, requiring absolute consistency in component parameters between both paths. However, intrinsic component tolerances inevitably cause deviations from nominal values. Simultaneously, capacitor dielectric-constant drift at high temperatures and transient resistor parameter jumps induced by acceleration shocks broaden tolerance bands [14]. This coupled effect of environmental stress and manufacturing variation disrupts dual-channel symmetry, invalidating common-mode rejection and degrading receiver SNR, necessitating rigorous circuit tolerance analysis [8,15,16,17]. Yet current tolerance analysis methods face significant limitations:

(1) The inability to pinpoint key contributors to performance fluctuations in multi-component circuits.
(2) Neglect of coupling effects obscuring nonlinear interactions [18,19].
(3) Empirical tolerance allocation mechanisms that trigger excessive utilization of premium-grade components, lacking quantitative cost–performance optimization frameworks.

These deficiencies—unidentified critical contributing factors, inadequate coupling interpretability, and insufficient economic orientation—significantly constrain the realization of high-reliability, low-cost fuze receivers.

This study establishes a dual-channel time-domain correlation differential model for UWB receivers and pioneers a hybrid sensitivity analysis (SA) strategy (Morris-LHS-Sobol) to construct a sensitivity-driven tolerance framework. This approach quantitatively resolves component–performance interactions and enables cost-optimized precision allocation. Compared with conventional tolerance methods [20,21,22], sensitivity analysis (SA), a systematic methodology quantifying model output responsiveness to input perturbations, offers a robust solution [9,23]. In circuit tolerance contexts, SA precisely identifies critical components and characterizes parameter interactions, particularly vital for nonlinear systems [24,25,26,27]. By ranking sensitivity indices, it guides tolerance allocation: imposing strict constraints on high-impact elements while relaxing standards for low-sensitivity components, co-optimizing performance stability, manufacturing cost, and production yield to establish a data-driven framework for high-reliability circuit design [28,29,30], ultimately achieving Pareto-optimal tradeoffs among performance robustness, cost-efficiency, and reliability metrics. Extensive circuit emulations validate the accuracy of the proposed methodology, while fuze receiver hardware measurements further confirm its significant enhancement of output stability, thereby establishing the theoretical foundation for robust tolerance design in high-frequency electronic systems.

The subsequent sections are structured as follows: Section 2 establishes the tolerance mathematical model for the UWB fuze dual-path differential receiver. Section 3 proposes a hybrid sensitivity strategy for tolerance assessment. Section 4 details physical simulation validation. Section 5 concludes with research outlooks.

## 2. Receiver Modeling Under Parameter Deviations

### 2.1. Operational Framework of Dual-Path Correlated Receiver

**Figure 2:**
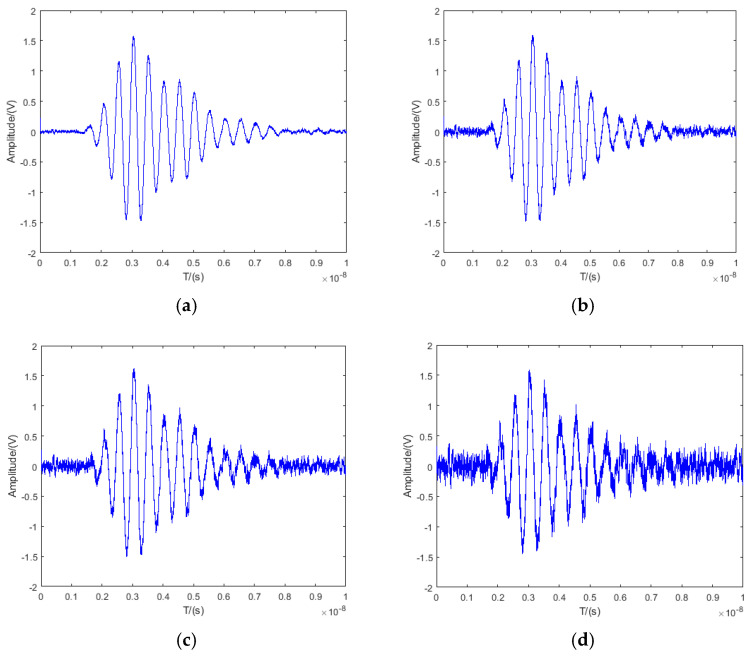
Output signal of dual-path time-domain correlation receiver: (**a**) $K_{1}-K_{2}=0$. (**b**) *K*_1_ − *K*_2_ = 0.01; (**c**) *K*_1_ − *K*_2_ = 0.05; (**d**) *K*_1_ − *K*_2_ = 0.1.

The dual-path time-domain correlation UWB fuze architecture in Figure 2, the echo signal, following reception by the antenna, is processed through a splitter and phase shifter, subsequently fed into the two channels, Correlation Receiver 1 and Correlation Receiver 2, with a phase difference of 180°. Under ideal conditions, the dual-path correlation receiver circuitry is symmetric, meaning both paths possess identical component parameter values [31]. Consequently, the input signals for the two paths $u_{1}(t)$ and $u_{2}(t)$ can be expressed, respectively, as follows:

(1) $$\left\{\begin{array}{l} u_{1}(t)=u_{r}(t)\;*\;h_{r}(t,\theta ,\phi )\\ u_{2}(t)=-u_{r}(t)\;*\;h_{r}(t,\theta ,\phi ) \end{array}\right.$$

where $u_{r}(t)$ represents the signal reflected from the target, $h_{r}(t,\theta ,\phi )$ denotes the unit impulse response of the fuze receiving antenna, and ∗ is the convolution operator.

Since the correlation receiver circuit essentially performs a correlation operation between the sampling pulse and the input signal of the correlation receiver, the signals reaching the differential amplifier are expressed as follows:

(2) $$\left\{\begin{array}{l} u_{out+}=s_{d}(t)\;*\;u_{1}(t)+n(r,c)\\ u_{out-}=s_{d}(t)\;*\;u_{2}(t)+n(r,c) \end{array}\right.,$$

where n(r,c) denotes the receiver-internal thermal noise, and $s_{d}(t)$ denotes the sampling pulse signal.

The correlated output of the ultra-wideband (UWB) radio fuze receiver is given by

(3) $$u_{0}(t)=A\times [u_{out+}-u_{out-}]=A\times [s_{d}(t)\;*\;u_{1}(t)-s_{d}(t)\;*\;u_{2}(t)]$$

where A denotes the gain of the differential amplifier. As evident from the above equation, the dual-path differential architecture effectively eliminates the internal thermal noise n(r,c), thereby reducing its impact on the receiver’s output signal and enhancing the system’s SNR.

However, in practical circuits, inherent component parameter variations preclude perfect symmetry between the two paths. Consequently, the above expression must be revised as follows:

(4) $$\begin{array}{cc} u_{0}(t) & =A\times [u_{out+}-u_{out-}]\\ & =A\times [s_{d}(t)\;*\;u_{1}(t)-s_{d}(t)\;*\;u_{2}(t)]+A\times (K_{1}-K_{2})\times n(r,c) \end{array}$$

In Expression (4), $K_{1}$ and $K_{2}$ represent the correlation-receiving gains of Correlation Receiver 1 and Correlation Receiver 2, respectively. As observed from the above formula, when the component parameters of Correlation Receiver 1 and Correlation Receiver 2 are perfectly symmetric (i.e., $K_{1}=K_{2}$), the receiver’s output signal contains no circuit thermal noise. However, due to the inherent inability to achieve perfect symmetry in dual-path component parameters, the circuit’s thermal noise cannot be fully eliminated, which can lead to a reduction in the output signal’s signal-to-noise ratio (SNR). Using MATLAB R2021a to simulate the above expression, the receiver’s output signals were analyzed under the following conditions: $K_{1}-K_{2}=0$, $K_{1}-K_{2}=0.01$, $K_{1}-K_{2}=0.05$, $K_{1}-K_{2}=0.1$.

The simulation results are illustrated in Figure 2.

The SNR of the output signal in the figure above is calculated as listed in Table 1.

As observed from Figure 2 and Table 1 above, for the dual-path time-domain correlation UWB fuze receiver under different gain deviations, a larger deviation magnitude correlates with a reduced SNR in the receiver’s output.

### 2.2. Receiver Model Based on Tolerance

**Figure 3:**
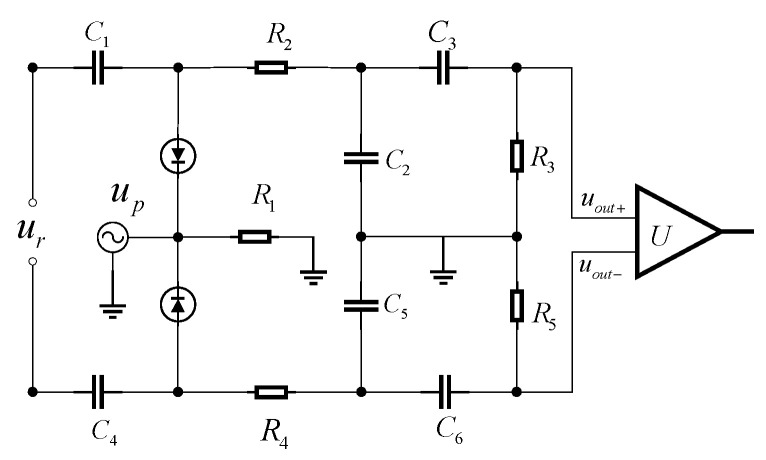
Balanced sampling integral differential circuit.

In the ultra-wideband radio fuze structure, the receiver is a crucial component of the fuze system, responsible for receiving signals reflected from the target [11]. The ultra-wideband fuze receiver adopts a sampling integration structure, which can reproduce periodic weak signals drowned in strong noise on one hand, and generate a distance gate using the sampling switch to only accept signals at a fixed distance on the other hand, thus achieving distance detection. Its circuit structure is shown in Figure 3.

**Figure 4:**
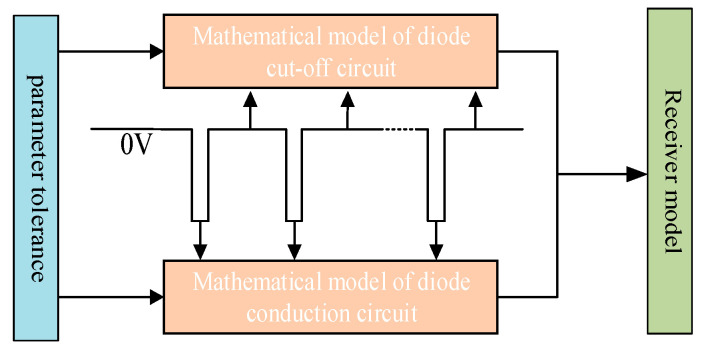
Tolerance-based mathematical model diagram.

Leveraging the bistable operational modes of the diode, the sampling–integration–differentiation circuit is partitioned into two distinct phases within a signal cycle: diode conduction and diode cutoff. A mathematical model is established for these two states to derive the expression for the output voltage. The schematic diagram of its principle is shown in Figure 4.

Since the upper and lower parts of the balanced sampling–integration–differentiation circuit operate in the same way, only the upper half of the circuit is considered here. The tolerance-based mathematical model aims to establish a sampling–integration–differentiation mathematical model under the condition of given tolerance ranges for circuit component parameters. This model calculates the impact of component parameter deviations on the circuit output and further analyzes the impact on circuit performance. Based on this, we can optimize our design and control the circuit error within a reliable range.

#### 2.2.1. Diode Conduction Mode Under Parameter Deviation

When the sampling pulse arrives, the diode conducts; the equivalent circuit is shown in Figure 4. The nominal values of the sampling capacitors, integrating capacitors, differentiating capacitors, integrating resistors, and differentiating resistors are $C_{1}$ and $C_{4}$, $C_{2}$ and $C_{5}$, $C_{3}$ and $C_{6}$, $R_{2}$ and $R_{4}$, and $R_{3}$ and $R_{5}$.

Due to component tolerances and external condition variations, the actual values become

(5) $$\left\{\begin{array}{l} C_{\delta 1}=C_{1}+\Delta \;C_{1}\\ C_{\delta 2}=C_{2}+\Delta \;C_{2}\\ C_{\delta 3}=C_{3}+\Delta \;C_{3}\\ R_{\delta 2}=R_{2}+\Delta \;R_{2}\\ R_{\delta 3}=R_{3}+\Delta \;R_{3} \end{array}\right.,\begin{array}{l} C_{\delta 4}=C_{4}+\Delta \;C_{4}\\ C_{\delta 5}=C_{5}+\Delta \;C_{5}\\ C_{\delta 6}=C_{6}+\Delta \;C_{6}\\ R_{\delta 4}=R_{4}+\Delta \;R_{4}\\ R_{\delta 5}=R_{5}+\Delta \;R_{5} \end{array},$$

where Δ denotes the relative offset from the nominal value.

Let the initial voltages across capacitors $C_{\delta 1}$ to $C_{\delta 6}$, before diode conduction in the n-th cycle be denoted as $u_{n1}$, $u_{n2}$, $u_{n3}$, $u_{n4}$, $u_{n5}$, and $u_{n6}$ respectively.

**Figure 5:**
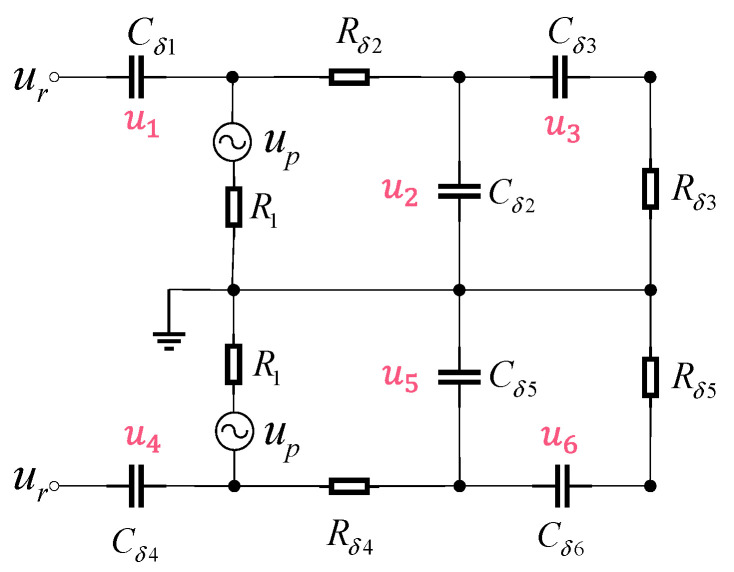
Equivalent circuit diagram during diode conduction.

Based on Figure 5, the following system of equations can be derived:

(6) $$\left\{\begin{array}{l} u_{r}=u_{1}+R_{1}(C_{\delta 1}\frac{du_{1}}{dt}-C_{\delta 2}\frac{du_{2}}{dt}-C_{\delta 2}\frac{du_{3}}{dt})+u_{p}\\ u_{2}=u_{r}-u_{1}-R_{\delta 2}(C_{\delta 2}\frac{du_{2}}{dt}+C_{\delta 3}\frac{du_{3}}{dt})\\ u_{3}=u_{2}-R_{\delta 3}C_{\delta 3}\frac{du_{3}}{dt}\\ u_{r}=u_{4}+R_{1}(C_{\delta 4}\frac{du_{4}}{dt}-C_{\delta 5}\frac{du_{5}}{dt}-C_{\delta 6}\frac{du_{6}}{dt})+u_{p}\\ u_{5}=u_{r}-u_{4}-R_{\delta 4}(C_{\delta 5}\frac{du_{5}}{dt}+C_{\delta 6}\frac{du_{6}}{dt})\\ u_{6}=u_{5}-R_{\delta 5}C_{\delta 6}\frac{du_{6}}{dt} \end{array}\right.,$$

In Equation (6), since $R_{3}\gg \;R_{2}\gg \;R_{1}$ and $R_{5}\gg \;R_{4}\gg \;R_{1}$, it follows that the current relationships satisfy $i_{C_{1}}\gg \;i_{C_{2}}\gg \;i_{C_{3}}$, $i_{C_{4}}\gg \;i_{C_{5}}\gg \;i_{C_{6}}$.

Equivalently, the capacitor dynamics exhibit

(7) $${C_{1}}^{\prime }\frac{du_{1}}{dt}\gg {C_{2}}^{\prime }\frac{du_{2}}{dt}\gg {C_{3}}^{\prime }\frac{du_{3}}{dt},\;{C_{4}}^{\prime }\frac{du_{4}}{dt}\gg {C_{5}}^{\prime }\frac{du_{5}}{dt}\gg {C_{6}}^{\prime }\frac{du_{6}}{dt},$$

To simplify solving the equation system [31], it is reformulated as follows:

(8) $$\left\{\begin{array}{l} u_{r}=u_{1}+R_{1}C_{\delta 1}\frac{du_{1}}{dt}+u_{p}\\ u_{2}=u_{r}-u_{1}-R_{\delta 2}C_{\delta 2}\frac{du_{2}}{dt}\\ u_{3}=u_{2}-R_{\delta 3}C_{\delta 3}\frac{du_{3}}{dt}\\ u_{r}=u_{4}+R_{1}C_{\delta 1}\frac{du_{1}}{dt}+u_{p}\\ u_{5}=u_{r}-u_{4}-R_{\delta 4}C_{\delta 5}\frac{du_{5}}{dt}\\ u_{6}=u_{5}-R_{\delta 5}C_{\delta 6}\frac{du_{6}}{dt} \end{array}\right.,$$

Applying the Laplace transform to Equation (8), it follows that

(9) $$\left\{\begin{array}{l} u_{r}/s=u_{1}(s)+R_{1}C_{\delta 1}[su_{1}(s)-u_{n1}]+u_{p}(s)\\ u_{2}(s)=u_{r}/s-u_{1}(s)+R_{\delta 2}C_{\delta 2}[su_{2}(s)-u_{n2}]\\ u_{3}(s)=u_{2}(s)-R_{\delta 3}C_{\delta 3}[su_{3}(s)-u_{n3}]\\ u_{r}/s=u_{4}(s)+R_{1}C_{\delta 4}[su_{4}(s)-u_{n4}]+u_{p}(s)\\ u_{5}(s)=u_{r}/s-u_{4}(s)-R_{\delta 4}C_{\delta 5}[su_{5}(s)-u_{n5}]\\ u_{6}(s)=u_{5}(s)-R_{\delta 5}C_{\delta 6}[su_{6}(s)-u_{n6}] \end{array}\right.,$$

Solving System (9) yields the following solution:

(10) $$\begin{array}{l} u_{1}(s)=\frac{(u_{n1}-u_{r}+u_{p})R_{1}C_{\delta 1}}{1+SR_{1}C_{\delta 1}}+\frac{u_{r}-u_{p}}{S}\\ u_{2}(s)=\frac{u_{n2}}{s+(1/R_{\delta 2}C_{\delta 2})}+u_{p}[\frac{1}{s}-\frac{1}{s+(1/R_{\delta 2}C_{\delta 2})}]-\frac{R_{1}C_{\delta 1}}{R_{1}C_{\delta 1}-R_{\delta 2}C_{\delta 2}}(u_{n1}-u_{r}+u_{p})[\frac{1}{s+(1/R_{1}C_{\delta 1})}-\frac{1}{s+(1/R_{\delta 2}C_{\delta 2})}]\\ u_{3}(s)=\frac{u_{n3}}{s+(1/R_{\delta 3}C_{\delta 3})}-\frac{1/R_{3}C_{\delta 3}}{s+(1/R_{\delta 3}C_{\delta 3})}[\frac{u_{n2}}{s+(1/R_{\delta 2}C_{\delta 2})}+u_{p}(\frac{1}{s}-\frac{1}{s+(1/R_{\delta 2}C_{\delta 2})})-\\ \;\;\;\;\;\;\;\;\;\;\;\;\;\;\;\frac{R_{1}C_{\delta 1}}{R_{1}C_{\delta 1}-R_{\delta 2}C_{\delta 2}}(u_{n1}-u_{r}+u_{p})\{\frac{1}{s+(1/R_{1}C_{\delta 1})}-\frac{1}{s+(1/R_{\delta 2}C_{\delta 2})}\}]\\ u_{4}(s)=\frac{(u_{n4}-u_{r}+u_{p})R_{1}C_{\delta 4}}{1+SR_{1}C_{\delta 4}}+\frac{u_{r}-u_{p}}{S}\\ u_{5}(s)=\frac{u_{n5}}{s+(1/R_{\delta 4}C_{\delta 5})}+u_{p}[\frac{1}{s}-\frac{1}{s+(1/R_{\delta 4}C_{\delta 5})}]-\frac{R_{1}C_{\delta 4}}{R_{1}C_{\delta 4}-R_{\delta 4}C_{\delta 5}}(u_{n4}-u_{r}+u_{p})[\frac{1}{s+(1/R_{1}C_{\delta 4})}-\frac{1}{s+(1/R_{\delta 4}C_{\delta 5})}]\\ u_{6}(s)=\frac{u_{n6}}{s+(1/R_{\delta 5}C_{\delta 6})}-\frac{1/R_{\delta 5}C_{\delta 6}}{s+(1/R_{\delta 5}C_{\delta 6})}[\frac{u_{n5}}{s+(1/R_{\delta 4}C_{\delta 5})}+u_{p}(\frac{1}{s}-\frac{1}{s+(1/R_{\delta 4}C_{\delta 5})})-\\ \;\;\;\;\;\;\;\;\;\;\frac{R_{1}C_{\delta 4}}{R_{1}C_{\delta 4}-R_{\delta 4}C_{\delta 5}}(u_{n4}-u_{r}+u_{p})\{\frac{1}{s+(1/R_{1}C_{\delta 4})}-\frac{1}{s+(1/R_{\delta 4}C_{\delta 5})}\}] \end{array},$$

Applying the inverse Laplace transform to Equation (10) yields

(11) $$\left\{\begin{array}{l} u_{1}(t)=\left(u_{n1}-u_{r}+u_{p}\right)e^{-\left(1/R_{1}C_{\delta 1}\right)t}+u_{r}-u_{p}\\ u_{2}(t)=u_{n2}e^{-\left(1/R_{\delta 2}C_{\delta 2}\right)t}+u_{p}\left(1-e^{-\left(1/R_{\delta 2}C_{\delta 2}\right)t}\right)-\frac{R_{1}C_{\delta 1}}{R_{1}C_{\delta 1}-R_{\delta 2}C_{\delta 2}}\left(u_{n1}-u_{r}+u_{p}\right)\left[e^{-\left(1/R_{1}C_{\delta 1}\right)t}-e^{-\left(1/R_{\delta 2}C_{\delta 2}\right)t}\right]\\ u_{3}(t)=u_{n3}e^{-\left(1/R_{\delta 3}C_{\delta 3}\right)t}-\frac{u_{n2}R_{\delta 2}C_{\delta 2}}{R_{\delta 2}C_{\delta 2}-R_{\delta 3}C_{\delta 3}}\left(e^{-\left(1/R_{\delta 2}C_{\delta 2}\right)t}-e^{-\left(1/R_{1}C_{\delta 1}\right)t}\right)+u_{p}\left(1-e^{-\left(1/R_{3}C_{\delta 3}\right)t}\right)-\\ \;\;\;\;\;\;\;\;\;\;\;\frac{u_{p}R_{\delta 2}C_{\delta 2}}{R_{\delta 2}C_{\delta 2}-R_{\delta 3}C_{\delta 3}}\left(e^{-\left(1/R_{\delta 2}C_{\delta 2}\right)t}-e^{-\left(1/R_{\delta 3}C_{\delta 3}\right)t}\right)-\frac{R_{1}C_{\delta 1}}{R_{1}C_{\delta 1}-R_{\delta 2}C_{\delta 2}}\left(u_{n1}-u_{r}+u_{p}\right)\\ \;\;\;\;\;\;\;\;\;\;\;\left[\frac{R_{1}C_{\delta 1}}{R_{1}C_{\delta 1}-R_{\delta 3}C_{\delta 3}}\left(e^{-\left(1/R_{1}C_{\delta 1}\right)t}-e^{-\left(1/R_{\delta 3}C_{\delta 3}\right)t}\right)--\frac{R_{\delta 2}C_{\delta 2}}{R_{\delta 2}C_{\delta 2}-R_{\delta 3}C_{\delta 3}}\left(e^{-\left(1/R_{\delta 2}C_{\delta 2}\right)t}-e^{-R_{\delta 3}C_{\delta 3}t}\right)\right]\\ u_{4}(t)=\left(u_{n4}-u_{r}+u_{p}\right)e^{-\left(1/R_{1}C_{\delta 4}\right)t}+u_{r}-u_{p}\\ u_{5}(t)=u_{n5}e^{-\left(1/R_{\delta 4}C_{\delta 5}\right)t}+u_{p}\left(1-e^{-\left(1/R_{\delta 4}C_{\delta 5}\right)t}\right)-\frac{R_{1}C_{\delta 4}}{R_{1}C_{\delta 4}-R_{\delta 4}C_{\delta 5}}\left(u_{n4}-u_{r}+u_{p}\right)\left[e^{-\left(1/R_{1}C_{\delta 4}\right)t}-e^{-\left(1/R_{\delta 4}C_{\delta 5}\right)t}\right]\\ u_{6}(t)=u_{n6}e^{-\left(1/R_{\delta 5}C_{\delta 6}\right)t}-\frac{u_{n5}R_{\delta 4}C_{\delta 5}}{R_{\delta 4}C_{\delta 5}-R_{\delta 5}C_{\delta 6}}\left(e^{-\left(1/R_{\delta 4}C_{\delta 5}\right)t}-e^{-\left(1/R_{\delta 5}C_{\delta 6}\right)t}\right)+u_{p}\left(1-e^{-\left(1/R_{\delta 5}C_{\delta 6}\right)t}\right)-\\ \;\;\;\;\;\;\;\;\;\;\;\frac{u_{p}R_{\delta 4}C_{\delta 5}}{R_{\delta 4}C_{\delta 5}-R_{\delta 5}C_{\delta 6}}\left(e^{-\left(1/R_{\delta 4}C_{\delta 5}\right)t}-e^{-\left(1/R_{5}^{\prime }C_{6}\right)t}\right)-\frac{R_{1}C_{\delta 4}}{R_{1}C_{\delta 4}-R_{\delta 4}C_{\delta 5}}\left(u_{n4}-u_{r}+u_{p}\right)\\ \;\;\;\;\;\;\;\;\;\;\;\left[\frac{R_{1}C_{\delta 4}}{R_{1}C_{\delta 4}-R_{\delta 5}C_{\delta 6}}\left(e^{-\left(1/R_{1}C_{\delta 4}\right)t}-e^{-\left(1/R_{\delta 5}C_{\delta 6}\right)t}\right)-\frac{R_{\delta 4}C_{\delta 5}}{R_{\delta 4}C_{\delta 5}-R_{\delta 5}C_{\delta 6}}\left(e^{-\left(1/R_{\delta 4}C_{\delta 5}\right)t}-e^{-R_{\delta 5}C_{\delta 6}t}\right)\right] \end{array}\right.,$$

#### 2.2.2. Diode Cutoff Mode Under Parameter Deviation

**Figure 6:**
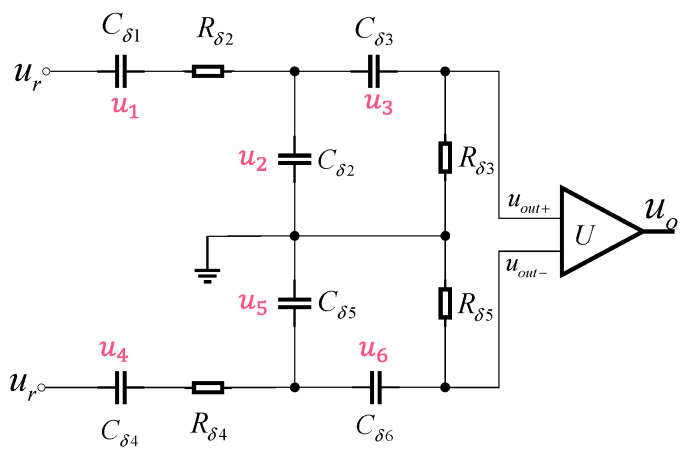
Equivalent circuit under diode cutoff condition.

Throughout the sampling pulse interval, the diode remains cutoff. The equivalent circuit during this phase is shown in Figure 6. At the start of the n-th cycle, when the diode enters the cutoff state, the initial voltages across capacitors $C_{\delta 1}$ to $C_{\delta 6}$ are denoted as $u_{n^{\prime }1}$, $u_{n^{\prime }2}$, $u_{n^{\prime }3}$, $u_{n^{\prime }4}$, $u_{n^{\prime }5}$, and $u_{n^{\prime }6}$ respectively.

Based on Figure 6, it follows that

(12) $$\left\{\begin{array}{l} u_{r}=R_{\delta 2}C_{\delta 1}\frac{du_{1}}{dt}+u_{1}+u_{2}\\ C_{\delta 1}\frac{du_{1}}{dt}=C_{\delta 2}\frac{du_{2}}{dt}+C_{\delta 3}\frac{du_{3}}{dt}\\ u_{2}=u_{3}+R_{\delta 3}C_{\delta 4}\frac{du_{3}}{dt}\\ u_{r}=R_{\delta 4}C_{\delta 4}\frac{du_{4}}{dt}+u_{4}+u_{5}\\ C_{\delta 4}\frac{du_{4}}{dt}=C_{\delta 5}\frac{du_{5}}{dt}+C_{\delta 6}\frac{du_{6}}{dt}\\ u_{5}=u_{6}+R_{\delta 5}C_{\delta 6}\frac{du_{6}}{dt}\\ u_{out+}=u_{2}-u_{3}\\ u_{out-}=u_{5}-u_{6}\\ u_{o}=A\;*\;(u_{out+}-u_{out-}) \end{array}\right.$$

Given $i_{C_{2}}\gg \;i_{C_{3}}$ and $i_{C_{5}}\gg \;i_{C_{6}}$, it follows that $C_{\delta 2}\frac{du_{2}}{dt}\gg C_{\delta 3}\frac{du_{3}}{dt}$ and $C_{\delta 5}\frac{du_{5}}{dt}\gg C_{\delta 6}\frac{du_{6}}{dt}$. System (12) is simplified as follows:

(13) $$\left\{\begin{array}{l} C_{\delta 1}\frac{du_{1}}{dt}=C_{\delta 2}\frac{du_{2}}{dt}\\ u_{r}=R_{\delta 2}C_{\delta 1}\frac{du_{1}}{dt}+u_{1}+u_{2}\\ u_{2}=u_{3}+R_{\delta 3}C_{\delta 3}\frac{du_{3}}{dt}\\ C_{\delta 4}\frac{du_{4}}{dt}=C_{\delta 5}\frac{du_{5}}{dt}\\ u_{r}=R_{\delta 4}C_{\delta 4}\frac{du_{4}}{dt}+u_{4}+u_{5}\\ u_{5}=u_{6}+R_{\delta 5}C_{\delta 6}\frac{du_{6}}{dt} \end{array}\right.$$

Applying the Laplace transform to Equation (13) yields

(14) $$\left\{\begin{array}{l} C_{\delta 1}[su_{1}(s)-u_{n^{\prime }1}]=C_{\delta 2}[su_{2}(s)-u_{n^{\prime }2}]\\ u_{r}/s=R_{\delta 2}C_{\delta 1}[su_{1}(s)-u_{n^{\prime }1}]+u_{1}(s)+u_{2}(s)\\ u_{2}(s)=u_{3}(s)+R_{\delta 3}C_{\delta 3}[su_{3}(s)-u_{n^{\prime }3}]\\ C_{\delta 4}[su_{4}(s)-{u_{n4}}^{\prime }]=C_{\delta 5}[su_{6}(s)-u_{n^{\prime }5}]\\ u_{r}/s=R_{\delta 4}C_{\delta 4}[su_{4}(s)-u_{n^{\prime }4}]+u_{4}(s)+u_{5}(s)\\ u_{5}(s)=u_{6}(s)+R_{\delta 5}C_{\delta 6}[su_{6}(s)-u_{n^{\prime }6}] \end{array}\right.,$$

Equation set (14) yields

(15) $$\left\{\begin{array}{l} u_{1}(s)=\frac{u_{n^{\prime }1}}{s+\left(1/R_{\delta 2}C_{\delta 2}\right)}-\frac{C_{\delta 2}\left(u_{n^{\prime }2}-u_{r}\right)-C_{\delta 1}u_{n^{\prime }1}}{C_{\delta 1}+C_{\delta 2}}\left[\frac{1}{s}-\frac{1}{s+\left(C_{\delta 1}+C_{\delta 2}\right)/R_{\delta 2}C_{\delta 1}C_{\delta 2}}\right]\\ u_{4}(s)=\frac{u_{n^{\prime }4}}{s+\left(1/R_{\delta 4}C_{\delta 5}\right)}-\frac{C_{\delta 5}\left(u_{n^{\prime }5}-u_{r}\right)-C_{\delta 4}u_{n^{\prime }4}}{C_{\delta 4}+C_{\delta 5}}\left[\frac{1}{s}-\frac{1}{s+\left(C_{\delta 4}+C_{\delta 5}\right)/R_{\delta 4}C_{\delta 4}C_{\delta 5}}\right] \end{array}\right.,$$

Performing the inverse Laplace transform on Equation (15) yields

(16) $$\left\{\begin{array}{l} u_{1}(t)=\frac{1}{C_{\delta 1}+C_{\delta 2}}[u_{n^{\prime }1}(C_{\delta 1}+C_{\delta 2}e^{-(C_{\delta 1}+C_{\delta 2})/R_{\delta 2}C_{\delta 1}C_{\delta 2}t})-(u_{n^{\prime }2}-u_{r})(C_{\delta 2}e^{-(C_{\delta 1}+C_{\delta 2})/R_{\delta 2}C_{\delta 1}C_{\delta 2}t}-C_{\delta 2})]\\ u_{4}(t)=\frac{1}{C_{\delta 4}+C_{\delta 5}}[u_{n^{\prime }4}(C_{\delta 4}+C_{5}e^{-(C_{\delta 4}+C_{\delta 5})/R_{\delta 4}C_{\delta 4}C_{\delta 5}t})-(u_{n^{\prime }5}-u_{r})(C_{\delta 4}e^{-(C_{\delta 4}+C_{\delta 5})/R_{\delta 4}C_{\delta 4}C_{\delta 5}t}-C_{\delta 5})] \end{array}\right.,$$

From the second and fifth equations of Equation Set (13), it follows that

(17) $$\left\{\begin{array}{l} u_{2}=u_{r}-u_{1}-R_{\delta 2}C_{\delta 1}\frac{du_{1}}{dt}\\ u_{5}=u_{r}-u_{4}-R_{\delta 4}C_{\delta 4}\frac{du_{4}}{dt} \end{array}\right.,$$

Differentiating Equation (16) yields

(18) $$\left\{\begin{array}{l} \frac{du_{1}}{dt}=-\frac{(u_{n^{\prime }1}+u_{n^{\prime }2}-u_{r})e^{-(C_{\delta 1}+C_{\delta 2})/R_{\delta 2}C_{\delta 1}C_{\delta 2}t}}{R_{\delta 2}C_{\delta 1}}\\ \frac{du_{4}}{dt}=-\frac{(u_{n^{\prime }4}+u_{n\prime 5}-u_{r})e^{-(C_{\delta 4}+C_{\delta 5})/R_{\delta 4}C_{\delta 4}C_{\delta 5}t}}{R_{\delta 4}C_{\delta 4}} \end{array}\right.,$$

Substituting Equation (18) into Equation (17) yields

(19) $$\left\{\begin{array}{l} u_{2}(t)=u_{n^{\prime }1}\frac{-C_{\delta 1}+C_{\delta 1}e^{-(C_{\delta 1}+C_{\delta 2})/R_{\delta 2}C_{\delta 1}C_{\delta 2}t}}{C_{\delta 1}+C_{\delta 2}}+(u_{n^{\prime }2}-u_{r})\frac{C_{\delta 2}+C_{\delta 1}e^{-(C_{\delta 1}+C_{\delta 2})/R_{\delta 2}C_{\delta 1}C_{\delta 2}t}}{C_{\delta 1}+C_{\delta 2}}+u_{r}\\ u_{5}(t)=u_{n^{\prime }4}\frac{-C_{\delta 4}+C_{\delta 4}e^{-(C_{\delta 4}+C_{\delta 5})/R_{\delta 4}C_{\delta 4}C_{\delta 5}t}}{C_{\delta 4}+C_{\delta 5}}+(u_{n^{\prime }5}-u_{r})\frac{C_{\delta 5}+C_{\delta 4}e^{-(C_{\delta 4}+C_{\delta 5})/R_{\delta 4}C_{\delta 4}C_{\delta 5}t}}{C_{\delta 4}+C_{\delta 5}}+u_{r} \end{array}\right.,$$

Applying the Laplace transform to Equation (19) gives

(20) $$\left\{\begin{array}{l} u_{2}(s)=\frac{u_{n^{\prime }1}C_{\delta 1}}{C_{\delta 1}+C_{\delta 2}}(\frac{1}{s}-\frac{1}{s+(C_{\delta 1}+C_{\delta 2})/R_{\delta 2}C_{\delta 1}C_{\delta 2}})+\frac{u_{r}}{s}+(u_{n^{\prime }2}-u_{r})[\frac{1}{s+(C_{\delta 1}+C_{\delta 2})/R_{\delta 2}C_{\delta 1}C_{\delta 2}}\\ \;\;\;\;\;\;\;\;\;\;+\frac{C_{\delta 2}}{C_{\delta 1}+C_{\delta 2}}(\frac{1}{s}-\frac{1}{s+(C_{\delta 1}+C_{\delta 2})/R_{\delta 2}C_{\delta 1}C_{\delta 2}})]\\ u_{5}(s)=\frac{u_{n^{\prime }4}C_{\delta 4}}{C_{\delta 4}+C_{\delta 5}}(\frac{1}{s}-\frac{1}{s+(C_{\delta 4}+C_{\delta 5})/R_{\delta 4}C_{\delta 4}C_{\delta 5}})+\frac{u_{r}}{s}+(u_{n^{\prime }5}-u_{r})[\frac{1}{s+(C_{\delta 4}+C_{\delta 5})/R_{\delta 4}C_{\delta 4}C_{\delta 5}}\\ \;\;\;\;\;\;\;\;\;\;+\frac{C_{\delta 5}}{C_{\delta 4}+C_{\delta 5}}(\frac{1}{s}-\frac{1}{s+(C_{\delta 4}+C_{\delta 5})/R_{\delta 4}C_{\delta 4}C_{\delta 5}})] \end{array}\right.,$$

From the third and sixth equations in Equation Set (14), it follows that

(21) $$\left\{\begin{array}{l} u_{3}(s)=\frac{u_{n^{\prime }3}+(1/R_{\delta 3}C_{\delta 3})u_{2}(s)}{s+1/R_{\delta 3}C_{\delta 3}}\\ u_{6}(s)=\frac{u_{n^{\prime }6}+(1/R_{\delta 5}C_{\delta 6})u_{2}(s)}{s+1/R_{\delta 5}C_{\delta 6}} \end{array}\right.,$$

The variables $\tau_{3}$ and $\tau_{6}$ are defined by the following expressions:

(22) $$\left\{\begin{array}{l} \tau_{3}=s+(C_{\delta 1}+C_{\delta 2})/R_{\delta 2}C_{\delta 1}C_{\delta 2}](s+1/R_{\delta 3}C_{\delta 3})\\ \tau_{6}=s+(C_{\delta 4}+C_{\delta 5})/R_{\delta 4}C_{\delta 4}C_{\delta 5}](s+1/R_{\delta 5}C_{\delta 6}) \end{array}\right.,$$

Substituting Equation (20) into Equation (21) leads to

(23) $$\left\{\begin{array}{l} u_{3}(s)=\frac{u_{n^{\prime }3}}{s+1/R_{\delta 3}C_{\delta 3}}-\frac{u_{n^{\prime }1}C_{\delta 1}}{C_{\delta 1}+C_{\delta 2}}[\frac{1/R_{\delta 3}C_{\delta 3}}{s(s+1/R_{\delta 3}C_{\delta 3})}-\frac{1}{R_{\delta 3}C_{\delta 3}\tau_{3}}]+\frac{(1/R_{\delta 3}C_{\delta 3})u_{r}}{s(s+1/R_{\delta 3}C_{\delta 3})}+\\ \;\;\;\;\;\;\;\;\;\;\;\;(u_{n^{\prime }2}-u_{r})\{\frac{1}{R_{\delta 3}C_{\delta 3}\tau_{3}}+\frac{C_{\delta 2}}{C_{\delta 1}+C_{\delta 2}}[\frac{1/R_{\delta 3}C_{\delta 3}}{s(s+1/R_{\delta 3}C_{\delta 3})}-\frac{1}{R_{\delta 3}C_{\delta 3}\tau_{3}}]\}\\ u_{6}(s)=\frac{u_{n^{\prime }6}}{s+1/R_{\delta 5}C_{\delta 6}}-\frac{u_{n^{\prime }4}C_{\delta 4}}{C_{\delta 4}+C_{\delta 5}}[\frac{1/R_{\delta 5}C_{\delta 6}}{s(s+1/R_{\delta 5}C_{\delta 6})}-\frac{1}{R_{\delta 5}C_{\delta 6}\tau_{6}}]+\frac{(1/R_{\delta 5}C_{\delta 6})u_{r}}{s(s+1/R_{\delta 5}C_{\delta 6})}+\\ \;\;\;\;\;\;\;\;\;\;\;\;(u_{n^{\prime }5}-u_{r})\{\frac{1}{R_{\delta 5}C_{\delta 6}\tau_{6}}+\frac{C_{\delta 5}}{C_{\delta 4}+C_{\delta 5}}[\frac{1/R_{\delta 5}C_{\delta 6}}{s(s+1/R_{\delta 5}C_{\delta 6})}-\frac{1}{R_{\delta 5}C_{\delta 6}\tau_{6}}]\} \end{array}\right.$$

The variables $\lambda_{3}$ and $\lambda_{6}$ are defined by the following expressions:

(24) $$\left\{\begin{array}{l} \lambda_{3}=\frac{1/R_{\delta 3}C_{\delta 3}}{1/R_{\delta 3}C_{\delta 3}-(C_{\delta 1}+C_{\delta 2})/R_{\delta 2}C_{\delta 1}C_{\delta 2}t}(e^{-(C_{\delta 1}+C_{\delta 2})/R_{\delta 2}C_{\delta 1}C_{\delta 2}t}-e^{-(1/R_{\delta 3}C_{\delta 3})t})\\ \lambda_{6}=\frac{1/R_{\delta 5}C_{\delta 6}}{1/R_{\delta 5}C_{\delta 6}-(C_{\delta 4}+C_{\delta 5})/R_{\delta 4}C_{\delta 4}C_{\delta 5}t}(e^{-(C_{\delta 4}+C_{\delta 5})/R_{\delta 4}C_{\delta 4}C_{\delta 5}t}-e^{-(1/R_{\delta 5}C_{\delta 6})t}) \end{array}\right.,$$

Performing the inverse Laplace transform on Equation (23) yields

(25) $$\left\{\begin{array}{l} u_{3}(t)=u_{n^{\prime }3}e^{-(1/R_{\delta 3}C_{\delta 3})t}-\frac{u_{n^{\prime }1}C_{\delta 1}}{C_{\delta 1}+C_{\delta 2}}[1-e^{-(1/R_{\delta 3}C_{\delta 3})t}-\lambda_{3}]+u_{r}(1-e^{-(1/R_{\delta 3}C_{\delta 3})t})+\\ \;\;\;\;\;\;\;\;\;\;\;\;\;\;\;\;(u_{n^{\prime }2}-u_{r})\{\lambda_{3}+\frac{C_{\delta 2}}{C_{\delta 1}+C_{\delta 2}}[1-e^{-(1/R_{\delta 3}C_{\delta 3})t}-\lambda_{3}]\}\\ u_{6}(t)=u_{n^{\prime }6}e^{-(1/R_{\delta 5}C_{\delta 6})t}-\frac{u_{n^{\prime }4}C_{\delta 4}}{C_{\delta 4}+C_{\delta 5}}[1-e^{-(1/R_{\delta 5}C_{\delta 6})t}-\lambda_{6}]+u_{r}(1-e^{-(1/R_{\delta 5}C_{\delta 6})t})+\\ \;\;\;\;\;\;\;\;\;\;\;\;\;\;\;\;(u_{n^{\prime }5}-u_{r})\{\lambda_{6}+\frac{C_{\delta 5}}{C_{\delta 4}+C_{\delta 5}}[1-e^{-(1/R_{\delta 5}C_{\delta 6})t}-\lambda_{6}]\} \end{array}\right.$$

To summarize, the subsequent stage to the balanced sampling–integration–differentiation circuit is a differential amplifier, which amplifies $u_{out+}-u_{out-}$ as the overall circuit output; A denotes the amplification factor of the differential amplifier. Through repeated iterations of the aforementioned simulation methodology, the receiver output $u_{o}$ is obtained as follows:

(26) $$u_{o}=A\;*\;(u_{out+}-u_{out-}),$$

where $u_{out+}$ and $u_{out-}$ are expressed as follows:

(27) $$\left\{\begin{array}{l} u_{out+}=u_{2}-u_{3}\\ u_{out-}=u_{5}-u_{6} \end{array}\right.,$$

The output voltage $u_{o}$ is obtained by substituting the previously determined variables $u_{2}$, $u_{3}$, $u_{5}$, and $u_{6}$ into Equations (26) and (27).

## 3. Sensitivity Analysis

Circuit performance metrics—such as noise suppression capability, gain, and linearity—are inevitably compromised by component parametric variations (e.g., resistor tolerances, amplifier offsets, temperature drift) and environmental perturbations. To quantitatively assess the impact of these parametric variations on critical receiver performance indicators and to optimize designs for enhanced robustness against parameter fluctuations, this paper proposes a dual-path correlation receiver tolerance modeling approach grounded in local and global sensitivity analysis theory. LSA will identify parameters most sensitive to performance near specific operating points, while GSA probes how parameter uncertainties and their interactions collectively contribute to system-level performance variability across broad parameter spaces.

This section defines target parameters and output variables for screening. By sampling parameters and conducting sensitivity analyses from both local and global perspectives, we identify high-sensitivity characteristic parameters requiring prioritized optimization in the dual-path time-domain correlation receiver model.

**Figure 7:**
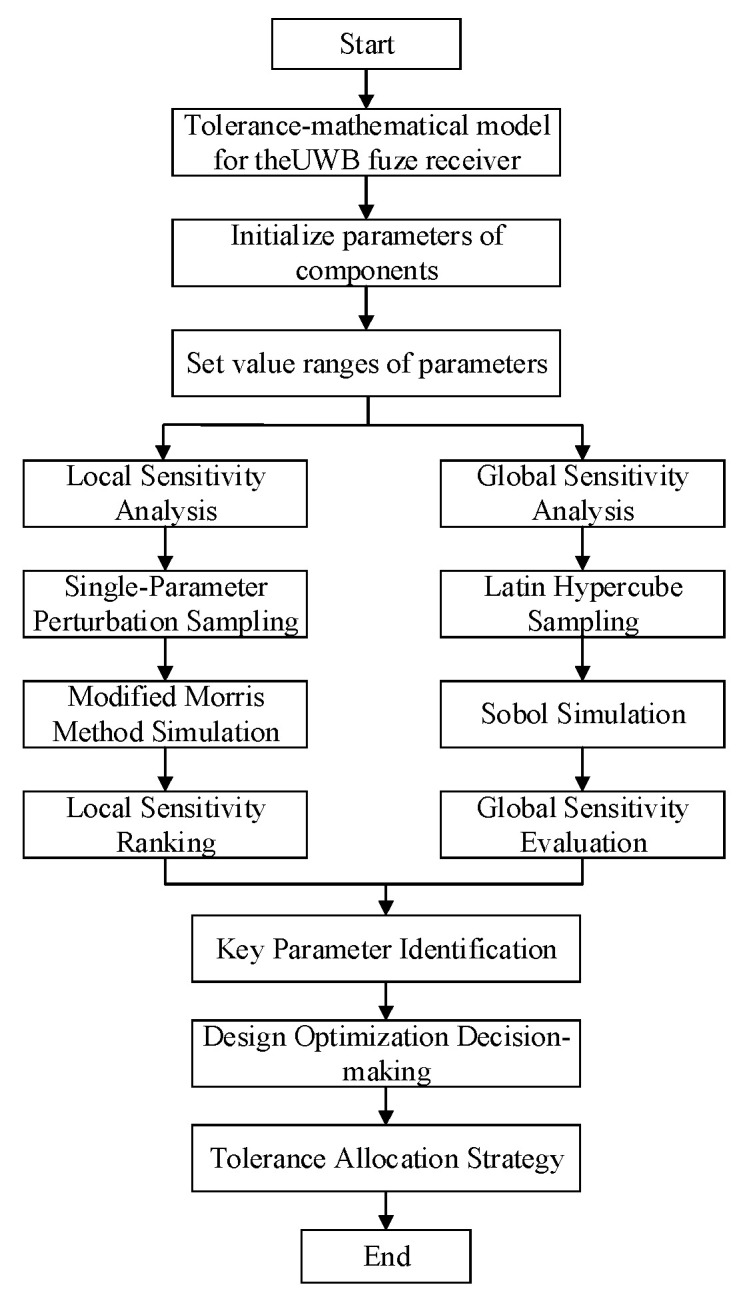
Sensitivity analysis workflow.

For this study, we employ the Modified Morris Screening Method and Partial Correlation Analysis (PCA) to conduct sensitivity analysis on parameters of the dual-path time-domain UWB fuze receiver model. Sensitivity analysis methods generally fall into two categories; the workflow is illustrated in Figure 7.

(1) Local Sensitivity Analysis (LSA)

LSA evaluates how minor perturbations in circuit parameters within the vicinity of nominal values affect output responses. It quantifies local gradients (e.g., first-order partial derivatives ∂Y/∂X_i_) to assess parametric sensitivity. Suitable for sensitivity evaluations at specific operating points, LSA rapidly identifies key parameters’ differential sensitivity coefficients. The widely adopted Morris Screening Method and its modified variant represent state-of-the-art LSA techniques.

(2) Global Sensitivity Analysis (GSA)

GSA methods quantify the aggregated statistical impact on output responses when multiple parameters vary concurrently across their spaces. They capture both independent parameter effects (main effects) and multi-parameter coupling effects (interactions), comprehensively reflecting parametric sensitivities.

### 3.1. Local Sensitivity Analysis Based on Modified Morris Screening Method

(a) Morris Local Sensitivity Analysis

The Morris Screening Method is a widely used approach for local sensitivity analysis of systems. Its core procedure involves selecting a specific component parameter $x_{i}$, applying a deviation Δx within its tolerance range while holding all other parameters constant, and executing the model to obtain the system output $u_{0}$:

(28) $$u_{0}\left(x\right)=U\left(x_{1},x_{2},x_{3},x_{4}\ldots .x_{n}\right)$$

The parameter’s impact on system output is then analyzed through

(29) $$S_{i}=\frac{{u_{0}}_{i}-u_{0}}{\Delta x}$$

where $u_{0}$ is the system output value before altering $x_{i}$;

Δx is the deviation magnitude applied to $x_{i}$;

${u_{0}}_{i}$ is the system output value after altering $x_{i}$.

The intrinsic randomness in variable selection within the standard Morris Screening Method necessitates the adoption of the Modified Morris Screening Method for evaluating parameter local sensitivity. This enhanced approach incorporates One-Factor-at-a-Time (OAT) sampling and delivers superior accuracy by introducing step-size-based perturbations to individual parameters while holding all other parameters fixed at nominal values. The resultant change in system output is systematically analyzed. The calculated sensitivity values are averaged to derive the parameter’s sensitivity coefficient, expressed as follows:

(30) $$S_{i}=\frac{1}{n-1}\sum_{i=0}^{n-1}\frac{({u_{0}}_{i+1}-{u_{0}}_{i})/u_{0}}{(P_{i+1}-P_{i})/100}$$

where n denotes the number of model runs.

${u_{0}}_{i+1}$ and ${u_{0}}_{i}$ represent the output values of the (*i* + 1)-th and *i*-th model runs, respectively.

$P_{i+1}$ and $P_{i}$ indicate the variation step sizes of parameters relative to their initial values during (*i* + 1)-th and *i*-th model runs.

**Figure 8:**
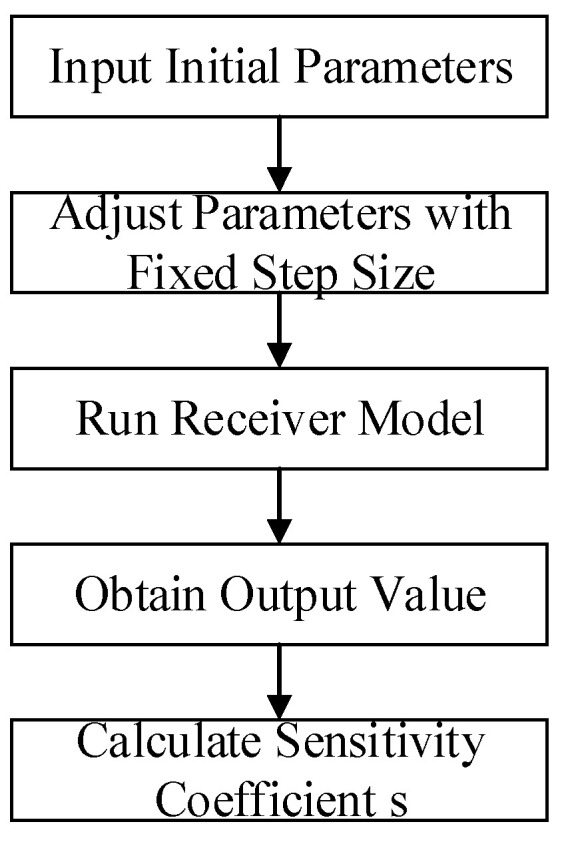
Modified Morris Screening Method analysis flow.

The dual-path time-domain correlation receiver primarily comprises resistors and capacitors, with surface-mount resistors typically featuring ±10% tolerance and ceramic capacitors exhibiting ±20% tolerance. To ensure parameter variations remain within reasonable bounds, we employ a fixed step size within the tolerance range for each analyzed parameter while holding all other parameters constant. These parameter values are then run in the model to analyze response characteristics under different parametric variations. The procedure is illustrated in Figure 8.

Parameters are classified by sensitivity level according to the criteria outlined in Table 2. A larger absolute value of the sensitivity coefficient ∣*S*∣ indicates a stronger influence of the parameter on the response variable. A positive *S* value signifies a positive correlation between the parameter and the output, while a negative *S* denotes an inverse relationship.

**Figure 9:**
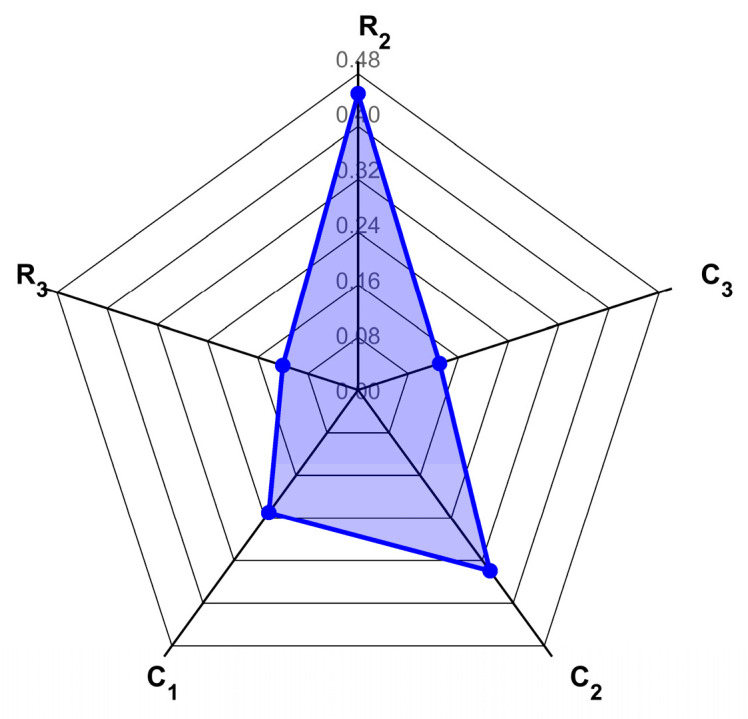
Single-parameter sensitivity simulation results.

The simulation results are shown in Figure 9.

Figure 9 shows that, compared to other components, the integrating capacitor *C*_2_ and integrating resistor *R*_2_ exhibit the highest sensitivity to the design objectives. These components play a significantly more pronounced role in regulating and optimizing the SNR and stability of the receiver’s output signal. Even minor adjustments to their parameters can trigger substantial variations in receiver output performance, thereby imposing a considerable impact on SNR.

(b) PDM Local Sensitivity Analysis

The Partial Derivative Method serves (PDM) as the fundamental approach for local sensitivity analysis, quantifying sensitivity by computing the partial derivative of model output $u_{o}$ with respect to input parameter x. Its mathematical definition is expressed as follows:

(31) $$S_{i}={\left.\frac{\partial u_{o}}{\partial x_{i}}\right|}_{x=x_{0}}$$

where $S_{i}$ denotes the sensitivity of output $u_{o}$ to the *i*-th input parameter $x_{i}$ at the baseline point $x_{0}$. Here, x represents components with i=1, 2, 3, 4, 5. The sensitivities for each component were computed by substituting parameters into the aforementioned Equation (31), with results benchmarked against the Morris method, as summarized in Table 3.

As evidenced in Table 3, the sensitivity coefficients for critical parameters exhibit substantial consensus between both methods. Minor discrepancies observed for sub-parameters stem from the Morris method’s capability to capture localized nonlinear effects, thus validating its robustness for comprehensive sensitivity characterization.

### 3.2. Global Sensitivity Analysis Based on LHS-Sobol Method

**Figure 10:**
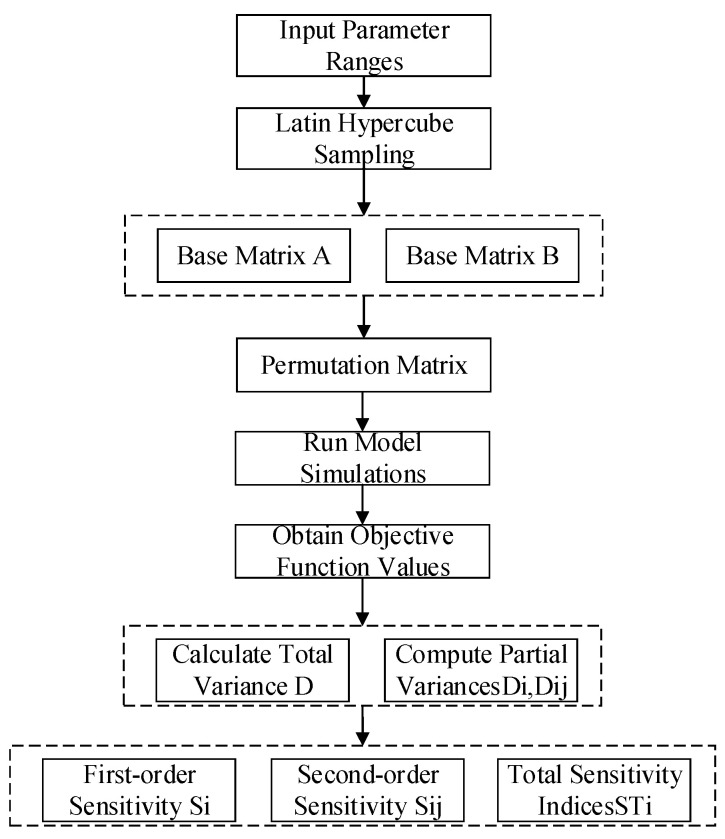
Sobol method analysis workflow.

The Sobol method constitutes a sensitivity analysis technique grounded in variance decomposition principles. As a global analysis approach, it effectively handles highly nonlinear and non-monotonic functions and models. By decomposing the total variance of the target function into contributions from individual parameters and their interactions, this method quantifies the influence of input parameters and parameter combinations on output variability. The process flow is illustrated in Figure 10.

As can be seen from Figure 10, the Sobol method requires extensive parameter sampling to ensure prediction accuracy, with computational results fundamentally dependent on the convergence and stability of the sampling technique [11]. Among diverse sampling strategies, the Latin Hypercube Sampling (LHS) method employs a stratified random sampling technique. Its core principle leverages the stratification of probability distributions, enabling more exhaustive exploration of the parameter space. This approach achieves precise reconstruction of probability distributions using fewer samples compared to conventional methods.

Latin Hypercube Sampling (LHS) Procedure:

(1) Determine Feature Dimension L: For the UWB fuze receiver output model, with 10 stochastic variables, $C_{\delta 1}$ to $C_{\delta 6}$ and $R_{\delta 2}$ to $R_{\delta 5}$, the feature dimension is L=10; an *L*-dimensional parameter space is constructed.

(2) Generate Sample Matrix: Assume sampling size N. Construct an N×L-dimensional sample matrix S.

(3) Stratified Sampling:

a. For each parameter $L_{k}(k=1,\ldots ,10)$, partition the probability space [0, 1] into N equisized intervals:

(32) $$\left[0,\frac{1}{N}\right),[\left.\frac{1}{N},\frac{2}{N}\right),\ldots ,[\frac{N-1}{N},1],$$

b. Within each interval, independently generate uniform random variates:

(33) $$u_{i}^{\left(k\right)}\sim u\left[\frac{i-1}{N},\frac{i}{N}\right],i=1,\ldots ,N,$$

yielding an N×11 stochastic matrix

(34) $$U=\left[\begin{array}{lll} u_{1}^{\left(1\right)} & \cdots & u_{1}^{\left(10\right)}\\ \vdots & \ddots & \vdots \\ u_{N}^{\left(1\right)} & \cdots & u_{N}^{\left(10\right)} \end{array}\right],$$

c. To suppress artificial spurious correlations between parameters, perform column-wise random permutation on U, ensuring independence of the sampled values.

(4) Inverse Cumulative Distribution Function (CDF) Transformation

Transform uniform samples U to the target distribution:

(35) $$S_{i,k}=F_{k}^{-1}(u_{i}^{\left(k\right)}),$$

where $F_{k}^{-1}(\cdot )$ is the inverse CDF of the k−th parameter. For a normal distribution, $F_{k}^{-1}(\cdot )$ is defined as follows:

(36) $$F_{k}^{-1}(u)=\mu_{k}+\sigma_{k}\cdot \Phi^{-1}(u),$$

with $\Phi^{-1}$ denoting the standard normal inverse CDF.

(5) Generation of Sample Matrices

The final N×L sample matrix is constructed as follows:

(37) $$A=\left[a_{1},a_{2}\ldots a_{L}\right]\left(L=10\right),$$

Each row represents a unique set of component parameters.

An independently generated base matrix B is created using the same sampling method. Both A and B are mutually independent. From these, permutation matrices C are derived as follows:

(38) $$\left\{\begin{array}{l} A=[a_{1},a_{2}\ldots a_{L}]\\ B=\left[b_{1},b_{2}\ldots b_{L}\right]\\ C^{\left(i\right)}=[a_{1}\cdots a_{i-1}b_{i}a_{i+1}\cdots a_{10}]\\ C^{\left(ij\right)}=[a_{1}\cdots b_{i}\cdots b_{j}\cdots a_{10}]\\ C^{\left(ijk\right)}=[a_{1}\cdots b_{i}\cdots b_{j}\cdots b_{k}\cdots a_{10}] \end{array}\right.,$$

The permutation matrices are constructed as follows:

$C^{\left(i\right)}$: Matrix formed by replacing the *i*-th column of A with the *i*-th column of B.

$C^{\left(ij\right)}$: Matrix formed by replacing the *i*-th and *j*-th columns of A with the corresponding columns of B, and so forth for higher-order interactions.

(a) Sobol Sensitivity Analysis

Sobol Sensitivity Analysis Procedure:

The UWB fuze receiver model is expressed as $y=u_{0}(x)$, where x denotes component parameter variables *x*_1_, *x*_2_, …, *x*_*n*_, and y represents the target variable. Substitute the sample matrix and permutation matrices x into the receiver model for simulation, yielding the following system outputs:

(39) $$\left\{\begin{array}{l} u_{A}=u_{0}(S)\\ u_{B}=u_{0}(S^{\prime })\\ u_{C^{\left(i\right)}}=u_{0}(C^{\left(i\right)})\\ u_{C^{\left(ij\right)}}=u_{0}(C^{\left(ij\right)})\\ u_{C^{\left(ijk\right)}}=u_{0}(C^{\left(ijk\right)}) \end{array}\right.,$$

The Sobol method, based on ANOVA variance decomposition principles, decomposes the output variance into contributions from input parameters and their interactions. For the circuit output y=u(x), the total variance decomposes as follows:

(40) $$\mathrm{Var}(y)=\sum_{i}V_{i}+\sum_{i<j}V_{ij}+\sum_{i<j<k}V_{ijk}+\cdots +V_{12\cdots p},$$

where

Var(y): Total model variance.

$V_{i}$: Variance contribution of *i-th* parameter.

$V_{ij}$: Interaction effect variance between parameters i and j.

$V_{12\cdots p}$: Variance from joint effects of p parameters.

First-order effect variance and first-order sensitivity index are given by

(41) $${\bar{V}}_{i}=\frac{1}{N}\sum_{s=1}^{N}u_{A}^{\left(s\right)}u_{C^{\left(i\right)}}^{\left(s\right)}-{\bar{u}}^{2},$$

(42) $${\hat{S}}_{i}=\frac{{\bar{V}}_{i}}{\bar{\mathrm{Var}}(u)},$$

Second-order effect variance and second-order sensitivity index are expressed as follows:

(43) $$\left\{\begin{array}{l} {\bar{V}}_{ij}=\frac{1}{N}\sum_{e=1}^{N}u_{A}^{\left(e\right)}u_{C^{\left(ij\right)}}^{\left(e\right)}-{\bar{V}}_{i}-{\bar{V}}_{j}-{\bar{u}}^{2}\\ {\hat{S}}_{ij}=\frac{{\bar{V}}_{ij}}{\mathrm{Var}(u)} \end{array}\right.,$$

Third-order and higher sensitivities follow by analogous extension. The total-effect sensitivity index is defined as follows:

(44) $$S_{T_{i}}=1-\frac{D_{∼i}}{D},$$

The first-order sensitivity index $S_{i}$ characterizes the effect of variations in a single parameter on the circuit output; the second-order sensitivity index $S_{ij}$ reflects the interaction effects when two parameters vary simultaneously; the total-effect index $S_{T_{i}}$ measures the comprehensive sensitivity of parameter *i*, incorporating both its isolated effect and all interactions with other parameters. $D_{∼i}$ denotes the variance from all parameters except parameter *i*.

**Figure 11:**
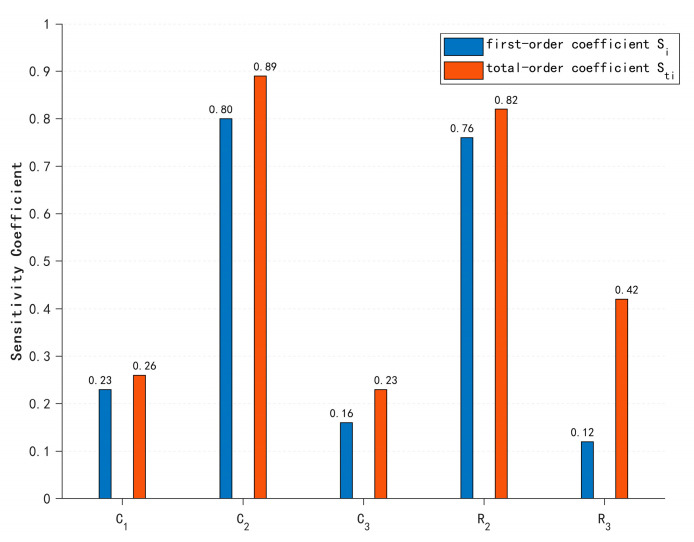
Comparative analysis of first-order and total-order sensitivity coefficients.

The calculated first-order and global sensitivity coefficients are presented as follows:

The computed second-order sensitivity indices are presented as follows:

Based on the Sobol method in global sensitivity analysis, first-order sensitivity indices (*S_i_*) quantify the independent contribution of individual input parameters to output variance. As evident from Figure 11, parameter *C*_2_ exhibits a significantly higher *Si* than other parameters, indicating its dominant individual impact on output variation. Parameter *R*_2_ also demonstrates substantial independent influence through its elevated *Si*. In contrast, *C*_3_ shows the lowest *S_i_*, revealing minimal independent contribution. This distribution highlights pronounced heterogeneity in parameter-level sensitivity magnitudes.

Critically, the combined first-order indices of *C*_2_ and *R*_2_ exceed unity, underscoring their collective indispensability within the model framework. The systematic dominance of total-effect indices $S_{ij}$ over $S_{i}$ (observed in the figure) confirms significant interaction effects. Core parameters *C*_2_ and *R*_2_ constitute a primary sensitivity axis characterized by positive synergistic effects. Parameter *R*_3_ displays enhanced interaction-driven sensitivity (*S_TR_*_3_ > *S_R_*_3_), signifying its role as a latent regulation factor via higher-order couplings—a hallmark of complex electronic systems.

**Figure 12:**
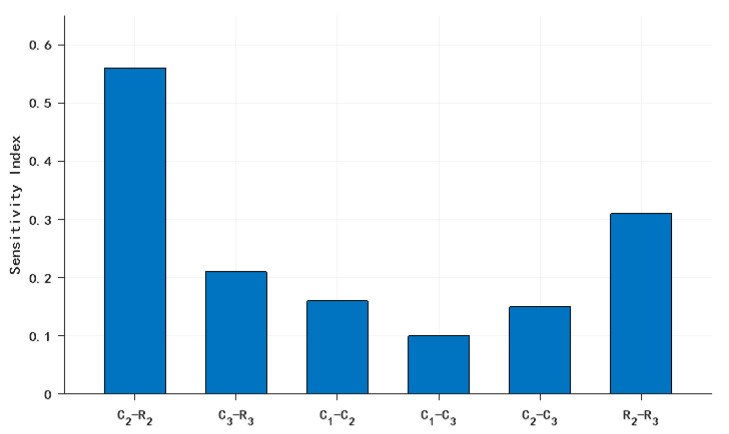
Comparative analysis of second-order sensitivity indices *S_ij_*.

Figure 12 shows that second-order interaction topology reveals marked heterogeneity: the pairwise interaction sensitivity index of the C_2_-R_2_ configuration exhibits dominant influence on the system-level response.

(b) Partial Rank Correlation Coefficient (PRCC) Sensitivity Analysis

**Figure 13:**
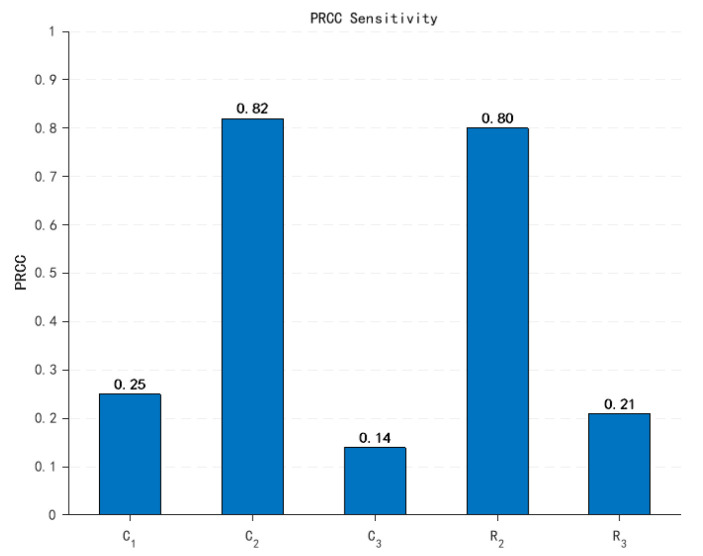
Sensitivity analysis results using PRCC.

The Partial Rank Correlation Coefficient (PRCC) method constitutes a non-parametric statistics-based approach for global sensitivity analysis. This technique operates by quantifying the strength of monotonic association between input parameters and output responses while controlling for confounding effects from other parameters, thereby evaluating the isolated main effect of individual parameters. Its mathematical formulation is expressed as follows:

(45) $${\mathrm{PRCC}}_{x_{j}}=\frac{\rho (r_{u},r_{x_{j}})-\rho (r_{u},r_{x_{-j}})\cdot \rho (r_{x_{j}},r_{x_{-j}})}{\sqrt{\left[1-\rho^{2}(r_{y},r_{x_{-j}})][1-\rho^{2}(r_{x_{j}},r_{x_{-j}})\right]}}$$

where ρ denotes the Spearman rank correlation coefficient, r represents the rank-transformed matrices of the samples, and x corresponds to components. Sensitivity analysis of the model via the PRCC method yields Partial Rank Correlation Coefficients, as summarized in Figure 13.

As evidenced in Figure 13, parameters $C_{2}$ and $R_{2}$ exhibit significantly stronger isolated effects on system output compared to other parameters. This result validates the reliability of the Sobol method in identifying critical parameters. However, it should be noted that PRCC solely quantifies individual parameter contributions and cannot resolve synergistic interactions between parameters. Consequently, this study employs the Sobol method for global sensitivity analysis to holistically quantify parameter influence mechanisms.

## 4. Physics-Based Modeling and Hardware Testing

Building upon the preceding analysis where we established a mathematical model for the dual-path time-domain UWB fuze receiver and evaluated the impact of individual component tolerances and multi-component tolerance interactions on receiver output, this section validates the model by constructing a circuit implementation in simulink. We systematically investigate the following:

(a) The effect of single-component tolerance variations on receiver output noise.
(b) The collective influence of stochastic multi-parameter variations on output noise performance.

### 4.1. Effect of Single Component on Circuit Performance

**Figure 14:**
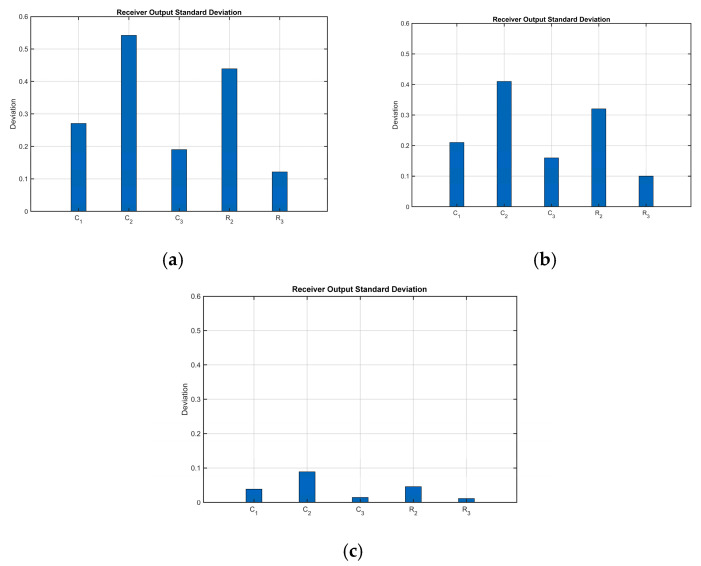
Output noise standard deviation at different tolerance: (**a**) deviations of 10%; (**b**) deviations of 5%; (**c**) deviations of 1%.

The standard deviations of receiver circuit output noise at individual component tolerance deviations of 1%, 5%, and 10% are as follows:

Figure 14 shows that the impact of individual components on receiver output noise was analyzed using standard deviation as the key quantitative metric. This systematic assessment reveals that compared to other components, the integrating capacitor and integrating resistor exert significantly more pronounced effects in regulating and optimizing the standard deviation and stability of the receiver’s output signal. Even minor adjustments to their parameters cause substantial changes in output noise levels, thereby considerably influencing SNR.

### 4.2. Impact of Full-Circuit Component Tolerances

**Figure 15:**
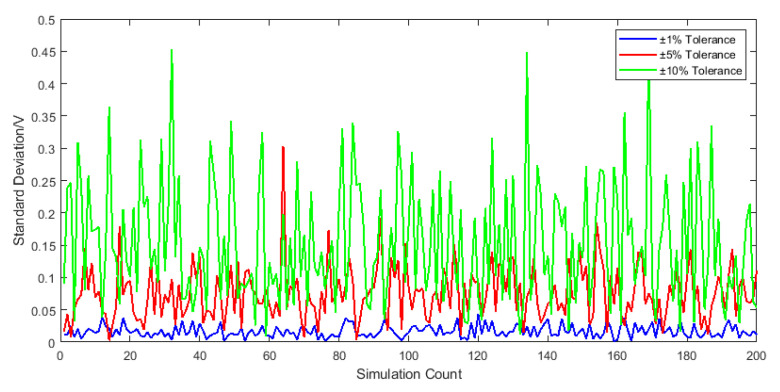
Output standard deviation distribution under randomized component variations.

To comprehensively evaluate the effects of parametric variations across all receiver components, stochastic tolerance assignments were applied within specified ranges. The simulation methodology and outcomes are detailed in Figure 15:

At ±10% tolerance levels, receiver output exhibits peak deviations of 450 mV. This magnitude of noise fluctuation poses significant interference risks to operational signals.

**Figure 16:**
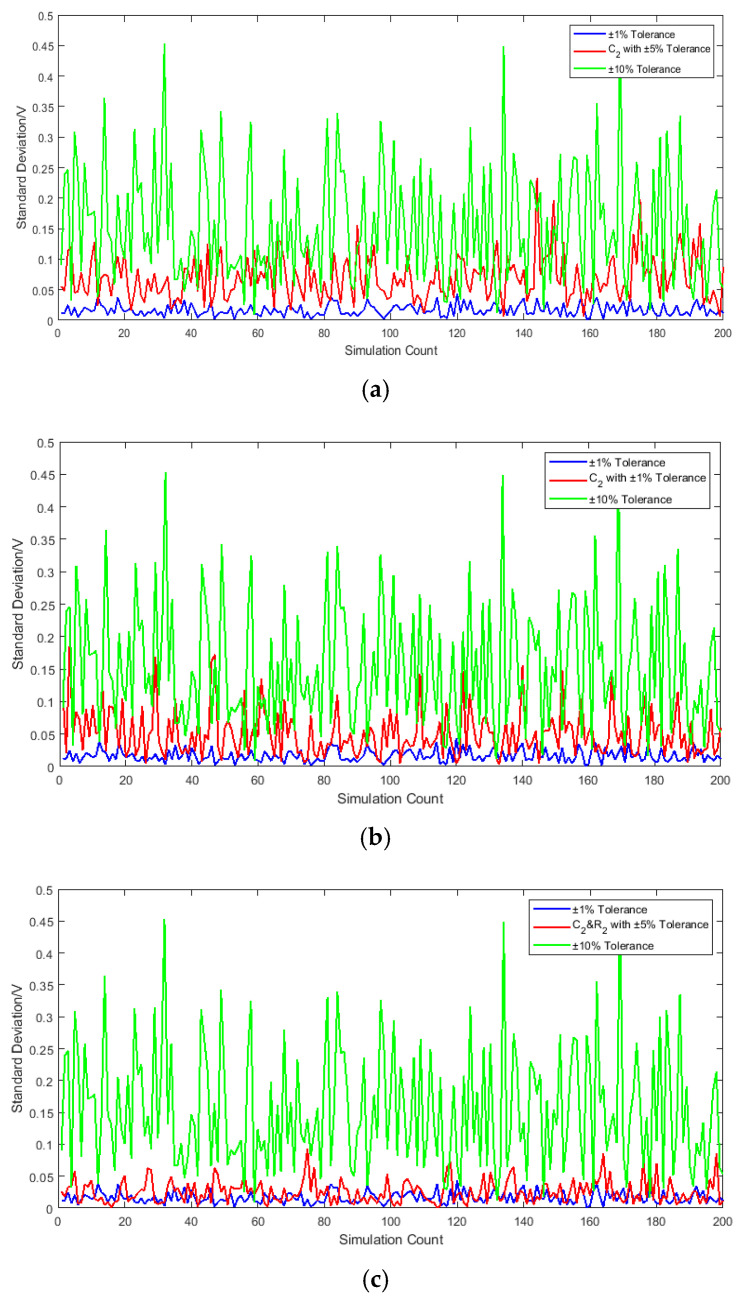
Effect of altering tolerance parameters of key components on system output standard deviation; (**a**) $C_{2}$ with 5% tolerance; (**b**) *C*_2_ with 1% tolerance; (**c**) *C*_2_ and *R*_2_ with 5% tolerance.

As established in prior analysis, different components exhibit distinct contributions to receiver output noise amplitude based on their deviation magnitudes. Specifically, sampling capacitor deviations contribute moderately, integrating resistor tolerances demonstrate higher impact, and integrating capacitor variations dominate. To minimize noise interference with operational signals while controlling component costs, precision enhancement of key circuit elements is implemented. By refining tolerances of the integrating capacitor, integrating resistor, and sampling capacitor to ±5%, the following standard deviation distributions are obtained through simulation:

The simulation result in Figure 16 demonstrates that enhancing the precision of the integrating capacitor $C_{2}$ and integrating resistor $R_{2}$ significantly reduces receiver output noise and improves SNR, as evidenced by the standard deviation distribution in the figure above. Concurrently, the simulation results reveal that the combinatorial effects of multiple component tolerances do not exhibit simple linear superposition. Under specific parametric combinations—even when individual component deviations remain within tolerance limits—performance degradation exceeding expectations was observed.

### 4.3. Experimental Hardware Verification

**Figure 17:**
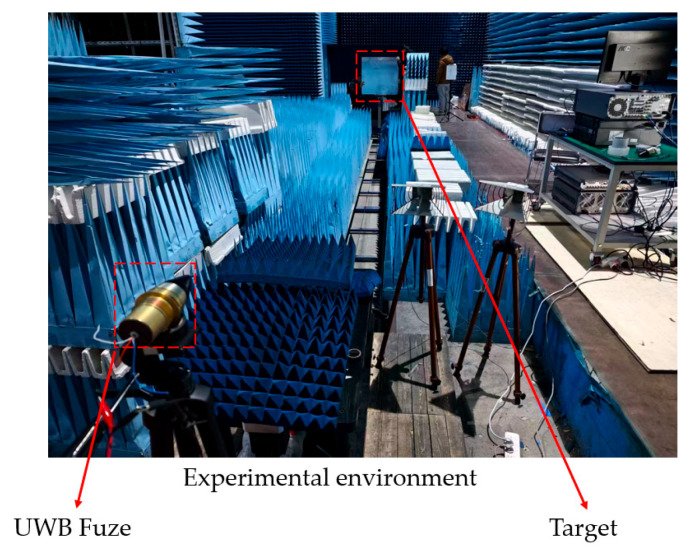
Test setup for UWB fuze prototype in EMI-shielded environment.

To validate simulation findings from the Morris-LHS-Sobol sensitivity-driven tolerance analysis model, this study conducted empirical validation on an ultra-wideband (UWB) fuze platform. Experiments employed three tolerance grades of integration capacitors and resistors (±1%, ±10%). Strict adherence to the single-variable control principle was ensured by using an identical fuze receiver platform, where parameter isolation was achieved through manual soldering replacement of capacitors with varying precision levels. To eliminate ambient electromagnetic interference, all tests were performed in a fully shielded microwave anechoic chamber, with the test environment depicted in Figure 17.

**Figure 18:**
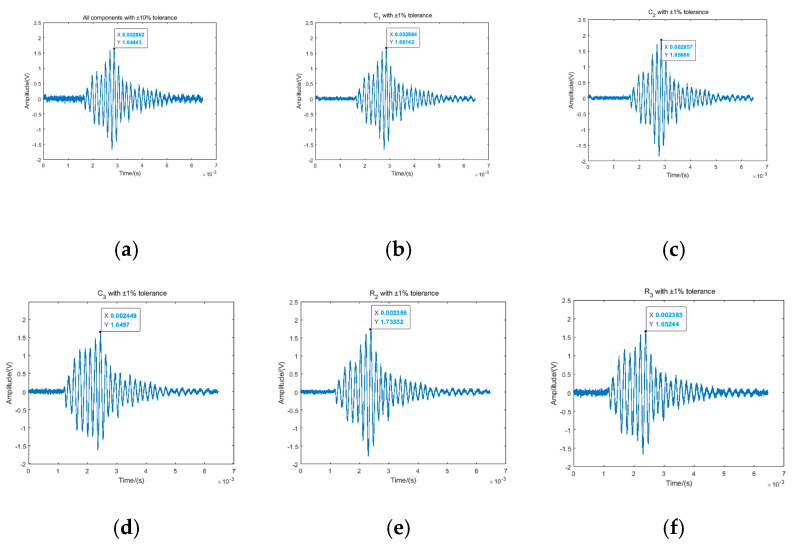
Output waveform of fuze receiver circuit: (**a**) tolerance of all components: ±10%; (**b**) *C*_1_ tolerance: ±11%; (**c**) *C*_2_ tolerance: ±1%; (**d**) *C*_3_ tolerance: ±1%; (**e**) *R*_2_ tolerance: ±1%; (**f**) *R*_3_ tolerance: ±1%.

The receiver was initialized with all components at ±10% tolerance, with its output signal recorded as the baseline. Sequentially, individual components were replaced with ±10% tolerance variants. To minimize experimental uncertainty, n = 10 repeated measurements were conducted per tolerance grade to ensure statistical significance, followed by signal-to-noise ratio (SNR) computation. This methodology was replicated to assess the performance improvement of C_1, C_2, C_3, R_2, and R_3 at ±1% tolerance on the fuze receiver. Resultant waveforms and SNR metrics are depicted in Figure 18 and Table 4, respectively.

Table 4 confirms strict adherence to single-variable control in the anechoic chamber using components with ±1% and ±10% tolerances for system validation. Initial SNR measurement with all ±10% components registered 15.63 dB. Precision soldering replacement of individual components to ±1% tolerance, coupled with n = 10 repeated measurements per configuration, yielded statistically significant improvements: Capacitor $C_{2}$ at ±1% tolerance increased SNR to 17.17 dB (Δ + 1.54 dB), Resistor $R_{2}$ to 17.01 dB (Δ + 1.38 dB), and Capacitor $C_{1}$ to 16.62 dB (Δ + 0.99 dB), while Capacitor $C_{3}$ and Resistor $R_{3}$ showed marginal gains at 16.19 dB (Δ + 0.56 dB) and 16.05 dB (Δ + 0.42 dB), respectively. This improvement hierarchy demonstrates marked consistency with the tolerance model’s sensitivity predictions, validating its engineering efficacy.

### 4.4. Comparison of Simulation Results

Section 3 and Section 4, respectively, employed mathematical modeling and circuit simulation to analyze the impact of component tolerance variations on receiver output, with the validity of the proposed methodology verified through physical testing of a fuzing prototype. The following conclusions can be drawn: Precision variation in the integrating capacitor *C*_2_ exerts a decisive influence on output voltage stability. When capacitor tolerance improved from 5% to 1%, the noise standard deviation decreased by 60%—precisely correlating with *C*_2_ high-sensitivity characteristics identified in sensitivity analysis. This confirms that *C*_2_ is the system’s pivotal sensitive element, whose precision enhancement directly and substantially improves SNR.

Resistor–Capacitor Synergy: Second-order sensitivity analysis revealed an interaction index of 0.56 for the *C*_2_-*R*_2_ pair, significantly exceeding other combinations, indicating strong parametric interactions. Physical simulations demonstrated optimal noise suppression (green curve) when concurrently enhancing *C*_2_ and *R*_2_ precision (1% tolerance for both), outperforming isolated *C*_2_ optimization (red curve). This validates the second-order sensitivity conclusion: *C*_2_-*R*_2_ interactions critically influence system performance.

Simulation and physical testing demonstrate that (1) refining *C*_2_ (high-sensitivity parameter) from 10% to 1% tolerance reduces noise by 60%; and (2) further noise reduction occurs when concurrently optimizing *R*_2_ (also highly sensitive). These results align with *C*_2_/*R*_2_’s elevated sensitivity coefficients and demonstrate their joint optimization efficacy via second-order interactions. Tolerance allocation optimization is proposed in Table 5.

This tolerance allocation framework achieves tri-objective optimization encompassing noise suppression, cost-efficiency, and manufacturing feasibility. Its core innovation lies in transforming Sobol global sensitivity analysis into actionable precision specifications, establishing a theoretically rigorous and practically implementable tolerance allocation paradigm for high-reliability electronic systems.

## 5. Conclusions

To address the challenge of internal thermal noise degradation in conventional single-channel time-domain correlated UWB fuze receivers, this study proposes a dual-channel time-domain correlated UWB fuze architecture. A dual-path asymmetric tolerance propagation model was established, and a Morris-LHS-Sobol hybrid sensitivity analysis framework was constructed to investigate the impact of component parameter deviations on system output. Rigorous physical simulations validated three principal findings:

First, the proposed dual-channel receiver achieves effective thermal noise suppression under ideal symmetry ($K_{1}=K_{2}$), yielding an SNR of 18.58 dB. Parameter deviations trigger progressive SNR degradation.

Second, the Morris-LHS-Sobol sensitivity methodology—first applied in fuze research—integrates two innovations: (i) combined LSA-GSA algorithms enabling rapid screening of local sensitivities and efficient identification of highly sensitive parameters; (ii) global exploration of multidimensional parameter spaces, comprehensively evaluating nonlinear interactions from component couplings and quantifying SNR–asymmetry correlations.

Third, analysis reveals significantly higher first-order sensitivity indices (*S_i_*) for parameters $R_{2}$ and $C_{2}$ versus others. Their pairwise second-order index (*S_ij_* = 0.56) confirms strong interactions, validated through physical simulations.

Beyond its application in the sensitivity analysis of component-level parametric tolerances within UWB fuze transmitters, the proposed methodology exhibits extensibility to reliability assessment across other critical subsystems, such as transmitter circuits. For cross-disciplinary scenarios, the proposed methodology enables precision-driven identification of reliability-critical components across broader electronic designs. By strategically prioritizing high-sensitivity elements while deprioritizing non-significant devices, it establishes a Pareto-optimized balance between reliability enhancement and cost reduction, preserving core performance metrics.

Moreover, the reliability of electronic systems is susceptible to extreme environments [32,33], owing to the inherent complexities of strongly nonlinear system responses, multiphysics field coupling, and microscopic material degradation mechanisms under such conditions. The current framework has not incorporated these extreme environmental factors. We have established a rigorous research trajectory integrating controlled experimentation and model iteration. Subsequent investigations will implement multiphysics-coupled experimentation under extreme operational regimes to quantify parametric interaction effects, thereby enhancing the engineering applicability and cross-platform generalizability of our analytical framework.

## Figures and Tables

**Table 1:** SNR of dual-path correlation receiver output under different gain deviations.

	Symmetry Ratio			
	$K_{1}-K_{2}=0$	$K_{1}-K_{2}=0.01$	$K_{1}-K_{2}=0.05$	$K_{1}-K_{2}=0.1$
SNR/dB	18.58	13.18	6.49	−2.16

**Table 2:** Parameter sensitivity classification criteria.

Class	Sensitivity Coefficient Range	Sensitivity Level
I	$1\leq \left|S\right|$	High Sensitivity
II	$0.3\leq \left|S\right|\leq 1$	Sensitive
III	$0.05\leq \left|S\right|\leq 0.3$	Moderate Sensitivity
IV	$0\leq \left|S\right|\leq 0.05$	Insensitive

**Table 3:** Parameter Sensitivity Analysis Comparison.

	Morris	PDM
$C_{1}$	0.23	0.21
$C_{2}$	0.46	0.48
$C_{3}$	0.13	0.16
$R_{2}$	0.35	0.32
$R_{3}$	0.12	0.11

**Table 4:** SNR of components at ±1% tolerance.

	10%Tolerance	$C_{1}$: ±1%	$C_{2}$: ±1%	$C_{3}$: ±1%	$R_{2}$: ±1%	$R_{3}$: ±1%
SNR/dB	15.63	16.62	17.17	16.19	17.01	16.05

**Table 5:** Tolerance allocation optimization.

Sensitivity Range	Components	Tolerance Strategy
Critical Sensitivity (S_Ti_ > 0.3)	*R*_2_, *C*_2_	±1% precision components
Moderate Sensitivity (0.2 < S_Ti_ < 0.3)	*C*_2_	±5% tolerance components
Low Sensitivity (S_Ti_ < 0.2)	*C*_1_, *R*_3_, *C*_3_	±10% commercial components

## Data Availability

The data are available from the corresponding author upon reasonable request.

## Abbreviations

The following abbreviations are used in this manuscript:

UWB	Ultra-Wideband
SNR	Signal-to-Noise Ratio
RF	Radio Frequency
MMW	Millimeter-Wave
SA	Sensitivity Analysis
GSA	Global Sensitivity Analysis
LSA	Local Sensitivity Analysis
PRCC	Partial Rank Correlation Coefficient
PDM	Partial Derivative Method
OAT	One-Factor-at-a-Time
LHS	Latin Hypercube Sampling
